# Melatonin: Awakening the Defense Mechanisms during Plant Oxidative Stress

**DOI:** 10.3390/plants9040407

**Published:** 2020-03-25

**Authors:** Adil Khan, Muhammad Numan, Abdul Latif Khan, In-Jung Lee, Muhammad Imran, Sajjad Asaf, Ahmed Al-Harrasi

**Affiliations:** 1 Natural and Medical Sciences Research Center, University of Nizwa, Nizwa 611, Oman; adilkhan@unizwa.edu.om (A.K.); numan2@unizwa.edu.om (M.N.); sajjadasaf@unizwa.edu.om (S.A.); 2 School of Applied Biosciences, Kyungpook National University, Daegu 41566, Korea; muhammad.imran@yahoo.com

**Keywords:** melatonin, ROS, antioxidant, ascorbate-glutathione cycle

## Abstract

Melatonin is a multifunctional signaling molecule that is ubiquitously distributed in different parts of a plant and responsible for stimulating several physio-chemical responses to adverse environmental conditions. In this review, we show that, although plants are able to biosynthesize melatonin, the exogenous application of melatonin to various crops can improve plant growth and development in response to various abiotic and biotic stresses (e.g., drought, unfavorable temperatures, high salinity, heavy metal contamination, acid rain, and combined stresses) by regulating antioxidant machinery of plants. Current knowledge suggests that exogenously applied melatonin can enhance the stress tolerance of plants by regulating both the enzymatic and non-enzymatic antioxidant defense systems. Enzymic antioxidants upregulated by exogenous melatonin include superoxide dismutase, catalase, glutathione peroxidase, and enzymes involved in the ascorbate–glutathione cycle (ascorbate peroxidase, monodehydroascorbate reductase, dehydroascorbate reductase, and glutathione reductase), whereas levels of non-enzymatic antioxidants such as ascorbate, reduced glutathione, carotenoids, tocopherols, and phenolics are also higher under stress conditions. The enhanced antioxidant system consequently exhibits lower lipid peroxidation and greater plasma membrane integrity when under stress. However, these responses vary greatly from crop to crop and depend on the intensity and type of stress, and most studies to date have been conducted under controlled conditions. This means that a wider range of crop field trials and detailed transcriptomic analysis are required to reveal the gene regulatory networks involved in the between melatonin, antioxidants, and abiotic stress.

## 1. Introduction 

Melatonin is a vertebrate pineal molecule that was first discovered in bovine pineal glands [1,2]. The pineal gland is responsible for producing melatonin to control behavioral responses to the photoperiod. It received its name after Lerner et al. [3] reported that it plays a role in lightening the skin color of frogs, while it is also involved in the control of circadian rhythms in various vertebrate species and acts as a neuronal protective antioxidant [4]. The highest levels of melatonin are recorded at night time, indicating that it is a major nocturnal signaling molecule [2]. Melatonin can be acquired through food in addition to being produced endogenously, though its rate of production declines with age [2,3,5].

In plants, the presence of melatonin has been confirmed in over 20 dicot and monocot families [6,7]. The presence of melatonin in different parts of a plant (e.g., roots, stems, leaves, fruit, flowers, and seeds) has been reported in various species such as onion, rice, tomato, banana, apple, and cucumber [7,8]. Melatonin has been associated with seed germination; fruit ripening; biomass production; photosynthesis; the circadian rhythm; membrane integrity; the redox network; root development; osmoregulation; leaf senescence; and responses to abiotic stresses such as drought, high salinity, heat, cold, oxidative stress, and heavy metal contamination [9,10,11,12,13,14,15]. In the last few years, the role of melatonin in the response to various abiotic and biotic stresses has been studied in detail [16]. Melatonin regulates the expression of genes known to aid in the protection of plants against environmental stresses [17]. Therefore, melatonin has been proposed as a bio-stimulant for sustainable crop production without negatively affecting the natural environment. 

Many researchers have studied various pathways that lead to melatonin formation and the scavenging of reactive oxygen species (ROS) when a plant is subject to abiotic stresses. On the basis of the results of past research, it can be concluded that there is a strong connection between ROS and melatonin within the antioxidant system. The available literature suggests that applying melatonin to crops that face abiotic stress regulates ROS levels, but the results have yet to be synthesized and analyzed in detail. Therefore, in this review, we seek to compile and review past findings on the regulation of antioxidant enzymes after the exogenous application of melatonin to various crops subject to abiotic stresses such as drought, heat, UV, high salinity, and heavy metal contamination (Table 1 and Table 2).

## 2. Biosynthesis of Melatonin in Plants

**Figure 1:**
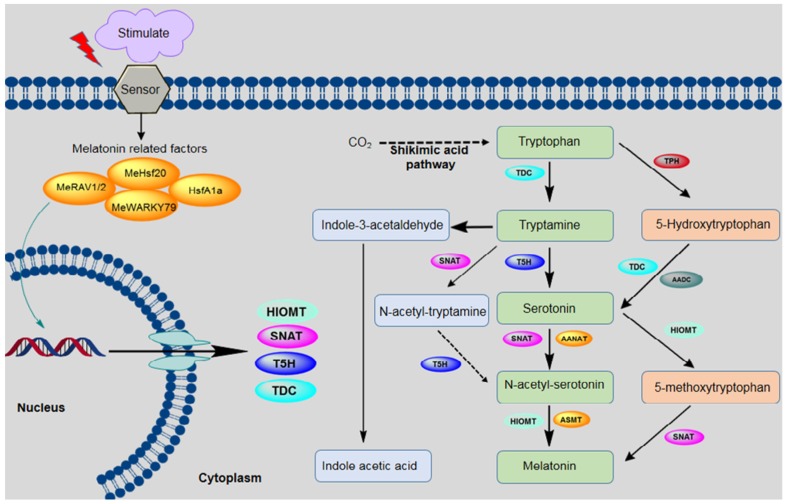
Biosynthetic pathway for melatonin in plants. The following enzymes are involved in this pathway: tryptophan decarboxylase (TDC), tryptamine 5-hydroxylase (T5H), arylalkylamine *N*-acetyltransferase (AANAT), serotonin-*N*-acetyltransferase (SNAT), *N*-acetylserotonin methyltransferase (ASMT), aromatic-L-amino-acid decarboxylase (AADC), hydroxyindole-O-methyltransferase (HIOMT), tryptophan hydroxylase (TPH), *N*-acetylserotonin deacetylase (ASDAC), and indole-3-acetic acid (IAA).

In the biosynthetic pathway for melatonin, the precursor tryptophan comes from synthesis via the shikimic acid pathway [7,53,54]. In addition to being the precursor for melatonin, tryptophan is also the precursor for indole-3-acetic acid (IAA) [55]. Tryptophan is converted into melatonin via four main enzymatic reactions, which are catalyzed by four different enzymes. Tryptophan decarboxylase (TDC) first converts tryptophan to tryptamine, and tryptamine 5-hydroxylase (T5H) then catalyzes the conversion of tryptamine to serotonin. These two steps are considered very important for serotonin synthesis in plants; however, another pathway also exists in some plants, in which tryptophan is converted by tryptophan 5-hydroxylase (TPH) into 5-hydroxytryptophan, which is then catalyzed by aromatic-L-amino-acid decarboxylase (TDC/AADC) into serotonin [56]. This alternative pathway is similar to the melatonin biosynthetic pathway that is observed in animals. In the third step, arylalkylamine *N*-acetyltransferase (AANAT) or *N*-acetyltransferase (SNAT) converts serotonin into *N*-acetyl-serotonin. SNAT can also convert tryptamine into *N*-acetyl-tryptamine, but T5H cannot convert *N*-acetyl-tryptamine into *N*-acetyl-serotonin. In the final step, *N*-acetyl-serotonin is catalyzed by *N*-acetyl-serotonin methyltransferase (ASMT) or hydroxyindole-O-methyltransferase (HIOMT) into melatonin. In addition to this main pathway, HIOMT can also convert serotonin into 5-methoxytryptamine, which is then converted into melatonin by SNAT [13,57] (Figure 1).

As shown in Figure 1, melatonin biosynthesis occurs by various pathways, and at least six enzymes are known to be involved. Interestingly, the accumulation of melatonin and its intermediate in various subcellular sites depend on the order of enzyme reactions involved in the biosynthesis of melatonin. For example, serotonin accumulation occurs in the endoplasmic reticulum when tryptophan is converted to serotonin by T5H, whereas in the case of TDC enzyme, serotonin accumulates in the cytoplasm. Similarly, serotonin is either converted to *N*-acetyl-serotonin or 5-methoxytryptamine by SNAT or ASMT, respectively. In the case of SNAT *N*-acetyl-serotonin accumulation occurred in chloroplast, whereas in the case of ASMT, the accumulation happens in the cytoplasm. Melatonin synthesis occurs in chloroplasts when the final-step enzyme is SNAT, whereas ASMT/Caffeic acid *O*-methyltransferase (COMT) are involved in the terminal reaction in the cytoplasm [58].

## 3. Reactive Oxygen Species (ROS) and the Plant Antioxidant Defense System 

An unavoidable consequence of aerobic metabolism is ROS production, including free radicals such as superoxide anions (O_2_^•−^) and hydroxyl radicals (^•^OH). Reactive oxygen species (ROS) can cause damage to plants (DNA damage, plant cell death), but also play an important role as signaling molecules that can help to regulate normal plant growth and response to different stresses. This also suggests the dual role of ROS in vivo depending on different levels of reactivity, potential, and productivity to cross the biological membrane [58]. Oxygen (O_2_), a source of all ROS, is stable and not very reactive in plants. However, it can be converted to high energy ROS in many organelles by various processes that can affect the plant’s metabolism [59]. ROS modifies and oxidizes the cellular components and prevents them from performing their original function [60]. Therefore, it is inevitable that evolution in the anoxygenic environment would necessitate the integration of oxidative processes and ROS sensing and signal into the developmental programs. From seed germination to plant senescence, ROS is dynamically generated or removed, which makes plants regulate their development to adapt to different environments. Under unfavorable circumstances, plants generate a large number of ROS species involved in the regulation of various processes including pathogen defense, programmed cell death (PCD), and stomatal behavior [61]. In plants, regarding reactive oxygen spices (ROS) produced in ionic and/or molecular form, ionic form has a superoxide anion (O.-^2^) and hydroxyl radical (·OH), whereas molecular form has a singlet oxygen (^1^O_2_) and hydrogen peroxide (H_2_O_2_) [59,60]. Every type of ROS has a different oxidative capacity to affect the different biochemical and physiological reaction, which is regulated by different genes in plants. Singlet oxygen excites oxygen generated in the chloroplast photosystem II (PS II), having a strong oxidizing ability and a very short life, being unstable once generated and having a greater impact on photosynthesis. Moreover, superoxide anion is the precursor of many ROS because it has instability and its strong oxidation and reducibility. Superoxide anion could maintain the stability of a plant stem cell [62]. Among these, H_2_O_2_ is considered an important redox molecule, given its specific physical and chemical properties, including remarkable stability within cells (half-life of 10^−3^ s), as well as rapid and reversible oxidation of target proteins [59]. Moreover, molecular oxygen is reduced in a series of steps following exposure to high energy and/or electron transfer. This leads to the production of highly reactive ROS. In plant species, ROS are formed via inevitable electron leakage onto O_2_ in the electron transport chain present in plasma membranes, chloroplasts, and mitochondria [63,64,65]. Environmental stresses such as drought, cold, high salinity, metal toxicity, and ultraviolet B (UV-B) radiation lead to greater ROS production in plants due to the disruption of cellular homeostasis [66,67,68,69,70,71]. When ROS levels are higher than what can be handled by the defense mechanisms, the cell experiences oxidative stress. Increased ROS production during periods of environmental stress poses a threat to cells due to lipid peroxidation, protein oxidation, nucleic acid damage, enzyme inhibition, and the activation of the programmed cell death pathway. All of these mechanisms can combine to eventually lead to cell death [67,72,73,74].

Though they can cause significant cellular damage, ROS molecules can also act as second messengers in many cellular processes [75,76]. Whether ROS molecules cause cellular damage or act a signaling molecule is dependent on the finely tuned equilibrium between ROS production and scavenging. As such, it is important to control ROS levels to avoid oxidative damage. The enzymatic and non-enzymatic antioxidant machinery of a plant plays a vital role in free radical scavenging [77]. Enzymatic antioxidants include superoxide dismutase (SOD); catalase (CAT); glutathione peroxidase (GPX); and enzymes in the ascorbate–glutathione (AsA-GSH) cycle such as ascorbate peroxidase (APX), monodehydroascorbate reductase (MDHAR), dehydroascorbate reductase (DHAR), and glutathione reductase (GR) [77]. AsA, GSH, carotenoids, tocopherols, and phenolics are potent non-enzymatic antioxidants in the cell. Several researchers have reported enhanced activity of the antioxidant machinery in plants to counter oxidative stress. Indeed, maintaining a strong antioxidant ability to scavenge toxic ROS molecules is associated with enhanced plant tolerance under harsh conditions [78,79].

## 4. Role of Endogenous Melatonin in the Plant Oxidative Stress System

The ability of melatonin to act as a scavenger of free hydroxyl radicals (OH^•^) and superoxide (O_2_^−^) was first recognized two decades ago [80]. It was found that physiological activity in plants can be enhanced by melatonin, which is an effective antioxidant. This molecule can move easily through cell membranes to the cytoplasm. Cells are separated from adjacent regions by the plasma membrane, which absorbs small molecules, but melatonin can easily pass through due to its amphipathic nature [13]. 

Following investigations into the role of endogenous melatonin in plants, it is now recognized as an ecofriendly molecule with a broad antioxidant ability [81,82]. Poeggeler et al. [83] also reported that melatonin is five times more active than GSH and 15 times more active than mannitol. In addition to having a direct role in scavenging many free radicals such as ROS and reactive nitrogen species (RNS), melatonin also acts as a signaling compound at the cellular level. Melatonin also upregulates several antioxidant enzymes, which increases its efficiency as an antioxidant [12,16,38].

Melatonin in plants shows that it functions as an effective antioxidant using both direct and indirect mechanisms. Melatonin signal transmission acts on ROS-mediated signals such as the balancing of hydrogen peroxide (H_2_O_2_), while also functioning as a direct antioxidant to maintain low cellular ROS levels. In fact, melatonin is more effective when it comes to reducing ROS levels than AsA. A large number of the metabolites that are produced during biosynthesis of melatonin, such as N1-acetyl-N2-formyl-5-methoxyknuramine (AFMK), cyclic 3-hydroxymelatonin (3-OHM), and 2-hyxdroxymelatonin are also influential antioxidants, promoting the antioxidant capabilities of this biomolecule [84] (Figure 1). In addition to that melatonin acts as a mediator in different antioxidant pathways, for example, the glutathione ascorbate cycle, peroxidases, superoxide dismutase, and catalase through varied mechanisms, resulting in abiotic and biotic stress responses in the plant [16]. 

Moreover, the literature has revealed that endogenous melatonin levels are higher under extreme environmental conditions. For example, Arnao and Hernández-Ruiz [85], Arnao and Hernández-Ruiz [86], and Byeon and Back [87] reported higher endogenous melatonin in the presence of stress. These studies have all suggested that stress can induce the biosynthesis of endogenous melatonin in plants. To conclude, the available literature indicates that melatonin is an effective mediator in the plant antioxidant system, resulting in abiotic and biotic stress responses in plants. 

## 5. Exogenous Melatonin with Plant Antioxidant Enzymes

Enzymatic antioxidants include CAT, SOD, GPX, and those involved in the AsA-GSH cycle, such as GR, MDHAR, APX, and DHAR. The effects of exogenous melatonin on each of these enzymes is discussed in this section.

### 5.1. Superoxide Dismutase (SOD)

SOD (E.C.1.15.1.1) belongs to the metalloenzyme family and represents the first line of defense against oxidative stress in all aerobic organisms [51,88]. SOD catalyzes the conversion of O_2_^•−^ to O_2_ and H_2_O_2_ [51]. SOD is found in the subcellular compartments that produce reactive oxygen. On the basis of its interaction with metal ions, SOD has been classified into three isozymes in plants—copper/zinc SOD, manganese SOD, and iron SOD—all of which are nuclear-encoded. FeSOD is found in chloroplasts whereas MnSOD is present in the mitochondria [66,89,90]. Copper/zinc SOD exists in three isoforms, localized in the cytosol, chloroplasts, peroxisome, and mitochondria [91,92,93]. 

SOD activity has been reported to be higher in plants exposed to various environmental stresses such as drought, high salinity, heat, and metal toxicity [8,13]. It has also been reported extensively in the literature that the exogenous application of melatonin further increases the activity of SOD enzymes under various environmentally harsh conditions such as metal toxicity [39], vanadium stress [47], high salinity [24], heat stress [37], cold stress [36], and drought [30]. The results of these studies indicate that the expression of SOD has a direct relation with exogenous melatonin levels. For example, Shi et al. [94] studied the effect of different concentrations of exogenous melatonin (4 µM, 20 µM, and 100 µM) on the activity of SOD under salt, drought, and cold stress. Their results indicated that melatonin had significant effects on SOD activity, alleviating abiotic stress-induced ROS accumulation and related oxidative damage in bermudagrass. Recently, Gao et al. [24] reported that the pre-treatment of tomato plants with different concentrations of melatonin (50 or 100 μM) significantly increased the activity of SOD under salinity stress. However, SOD activity in melatonin-treated naked oat seedlings was lower than in non-melatonin treated seedlings. Furthermore, several other researchers have also reported increased SOD activity during salinity stress, including Martinez, et al. [50], Chen, et al. [22], and Zhan, et al. [95], thus promoting plant growth.

Similarly, greater SOD activity has been reported during chilling stress [42], waterlogging [44], alkaline stress [46], heavy metal stress [38], and drought stress [26]. Campos et al. [25] reported that SOD activity was lower following the rehydration of *Coffea arabica* L. plants that had initially been exposed to drought stress, whereas Hodžić et al. [40] described changes in SOD activity following the addition of melatonin under Cd and Zn stress in *Melissa officinalis* L. and *Valeriana officinalis* L. Interestingly, lower SOD activity was observed in *Valeriana officinalis* L. supplemented with both Zn and melatonin, whereas it increased when Zn was replaced with Cd. However, in the case of *Melissa officinalis* L., higher SOD activity was recorded after the addition of melatonin with Cd and Zn. Thus, it can be concluded that melatonin pre-treatment enhances SOD activity, which consequently increases the tolerance of crop plants to various abiotic stresses. 

The details of the processes involved in the regulation of antioxidant enzymes by melatonin have not yet been reported. However, various experiments have observed gene expression occurring at the nanomolar level in melatonin-treated cells [96]. To date, various studies of plants have reported the upregulation of different SOD isoforms (i.e., ZnSOD and FeSOD) following treatment with exogenous melatonin [21,38,47,50,52,96]. Martinez et al. [50] found that the application of melatonin during combined stress (high salinity and heat) induced the greater expression of SOD (Cu/ZnSOD, FeSOD) compared to the control plants. 

The current literature suggests that, under harsh conditions, the activity of SOD increases with the application of exogenous melatonin. However, the complexity of SOD activity requires basic research to continue because SOD activity under abiotic stress differs in terms of its dependence on various factors such as plant species, plant parts, the concentration applied, and the duration of exposure. 

### 5.2. Catalase (CAT)

CAT (EC 1.11.1.6) is a tetrameric enzyme containing heme molecules that are found primarily in peroxisomes and that plays a role in catalyzing the dismutation of H_2_O_2_ molecules into O_2_ and 2H_2_O. CAT shows a strong preference for H_2_O_2_ molecules over organic peroxides. CAT is an independent antioxidant enzyme that does not require any reducing equivalent in the cellular compartments. The turnover rate of CAT is very high (6 × 10^6^ molecules of H_2_O_2_ to H_2_O and O_2_ min^−1^); however, it has a lower affinity for peroxide than does APX [51,97,98]. Peroxisomes are thought to be the main sites for the production of H_2_O_2_ resulting from β-oxidation, the catabolism of purines, oxidative stress, and photorespiration. However, recent reports have suggested that CAT is present in the cytosol, mitochondria, and chloroplasts [99]. The angiosperm species have been studied and are reported to have three CAT genes. Willekens et al. [100] proposed a classification system for CAT enzymes on the basis of expression profiling of the genes in tobacco. The CAT1 gene is coded in seeds and pollen, with these enzymes localized mainly in the cytosol and peroxisomes, whereas CAT2 is expressed mainly in the photosynthetic organs, in addition to the seeds and roots, and CAT3 is expressed in the vascular tissue and leaves and is primarily localized in the mitochondria. 

CAT mostly responds to various abiotic stresses via overproduction, which helps plants to maintain appropriate ROS levels. However, several studies have reported either an increase or decrease in CAT activity depending on the duration, intensity, and type of abiotic stress [67,69,101]. The effect of exogenous melatonin on the activity of CAT under abiotic stress conditions has also been investigated, with findings suggesting that it varies (i.e., increases, decreases, or does not affect CAT activity) under different abiotic stress conditions. For example, Chen et al. [22] reported that the application of exogenous melatonin (20 and 100 μM) significantly enhanced the activity of CAT in *Zea mays* L. under salinity stress. Similarly, Das and Roychoudhury [51] reported that the application of melatonin (300 mM) raised CAT levels in *Cynodon dactylon* L. under salinity stress. In particular, they suggested that exogenous melatonin alleviates the cell damage that occurs due to high levels of ROS production during salinity stress by increasing the activity of CAT. Indeed, Zheng et al. [44] found that melatonin treatment significantly enhanced the biosynthesis of CAT, which had been suppressed by waterlogging stress. Specifically, they reported that melatonin spray (200 μM) recovered the activity of CAT in leaves to levels comparable to the control seedlings. Higher levels of CAT under drought stress were also reported by Campos et al. [25], and similar effects between melatonin and CAT have been observed under acid rain stress [42], cold stress [33], and vanadium stress [47]. Several studies have also reported that melatonin treatment increases CAT activity under combined stresses such as salinity and heat stress [50]; salinity, drought, and cold stress [51]; and lead and acid rain stress [49]. However, Ni et al. [38] noted that CAT activity fell upon the application of melatonin (100 μM) in wheat seedlings under cadmium stress. Furthermore, Turk et al. [32] observed that wheat seedlings exposed to chilling stress exhibited higher levels of SOD, GPX, APX, and GR, but CAT activity was unchanged. Marta et al. [34] later reported a similar result in *Cucumis sativus* L. under chilling stress. 

To validate the influence of melatonin on CAT activity under various harsh conditions, several studies have investigated CAT gene expression, with findings similar to those observed for the levels of functional protein within the cell. Zhang et al. [21] reported that, under high salinity stress (150 mM), the application of exogenous melatonin (1 μM) significantly increased the expression of the CAT gene in *Cucumis sativus* L. Similarly, Nawaz et al. [47], Ni et al. [38], and Cao et al. [52] reported the higher expression of the CAT gene under vanadium and oxidative stress. However, under combined stresses such as salinity and heat stress, gene expression was downregulated in melatonin-treated plants (100 μM), as reported by Martinez et al. [50] in *Solanum lycopersicon* L. These findings suggest that melatonin enhances the expression of CAT genes depending on the stress type, duration, and intensity, helping plants to cope with abiotic stress.

From these studies, we can conclude that the exogenous use of melatonin on different crop species under various abiotic stresses generally increases CAT activity. However, as described above, several studies have reported the opposite results. These results suggest that CAT occasionally does not show any response to treatment with melatonin, as also described by [38]. Further studies are thus needed to clarify the interactive effects of melatonin on CAT activity under abiotic stresses.

### 5.3. Glutathione Peroxidase (GPX)

GPX (EC 1.11.1.9) is a heme-containing protein composed of 40–50 kDa monomers. It is widely found in animals, plants, and microbes. Many GPX isoenzymes exist in plant tissue localized in vacuoles, the cell wall, and the cytosol [51,102]. GPX is widely recognized as a stress enzyme, functioning as an effective quencher of reactive intermediary forms of O_2_ and peroxy radicals under conditions of stress [103]. Glutathione peroxidase is a family of many isozymes that can catalyze the reduction of H_2_O_2_ and cytotoxic hydrogen-peroxide to alcohols [104]. Besides GPX’s ability to scavenge H_2_O_2_, it also detoxifies the production of lipid-peroxidation, which form due to the activity of reactive oxygen spices (ROS). Glutathione peroxidase was classified into three types, glutathione transferases GPX activity (GST-GPX), selenium-dependent, and the non-selenium-dependent phospholipid hydroperxide (PHGPX). GPX eradicates peroxide as potential substrates for Fenton reaction. GPX also acts within conjunction with tripeptide glutathione (GSH), which is present in high amounts in the cells. The substrate for the catalytic reaction of GPX is H_2_O_2_ or an organic peroxide ROOH. Moreover, glutathione peroxidase decomposes the peroxides with water or alcohol during oxidization of GSH. GPX competes with catalase for H_2_O_2_ as a substrate and is the major source of protection against low levels of oxidative stress [105]. 

Under harsh conditions, the upregulation of components of the antioxidant system occurs at both the transcriptional and post-transcriptional levels. Of the various antioxidant enzymes, GPX and CAT represent the principal ROS scavengers in plants. In recent years, increasing evidence has been found that GPX in plants plays an important role in regulating plant responses to various abiotic stresses such as salt, drought, heat, cold, and oxidative stress [106,107,108,109,110,111]. In most cases, as with other antioxidant enzymes, the levels of functional GPX in the cell increases consistently. Several studies have consequently described the effect of exogenous melatonin on the activity of GPX under various abiotic stress conditions, with melatonin typically upregulating GPX levels in plants by regulating antioxidant-related gene expression. Recently, Chen et al. [22] demonstrated that the exogenous application of melatonin (20 and 100 μM) under salinity stress significantly increased the activity of GPX in *Zea mays* L.; however, this study also suggested that, under normal conditions, the application of melatonin (20 and 100 μM) did not increase the levels of functional GPX at the cellular level. Turk et al. [32] subsequently reported that the application of melatonin (1 mM) also increased the activity of GPX in *Tiritucum aestivum* exposed to cold stress. Recently, Huang et al. [31] described the effects of exogenous melatonin on cellular GPX activity. They noted that, under drought stress, GPX activity was lower in the roots but higher in the shoots, whereas exogenous melatonin increased GPX activity in the roots and decreased activity in the shoots. Furthermore, Martinez et al. [50] reported greater GPX activity in *Solanum lycopersicon* pre-treated with melatonin (100 μM) under combined stress (salinity + heat; 35 °C + 75 mM NaCl). 

With the aim to fill some of the gaps in the knowledge regarding the specific role of melatonin in the regulation of GPX at the mRNA level under various stresses, a number of studies have investigated GPX transcript levels. Martinez et al. [50] noted the overexpression of the GPX gene responsible for maintaining low levels of peroxidase in *Solanum lycopersicon* L. treated with exogenous melatonin (100 μM) exposed to high salt levels and heat, suggesting melatonin has a key role in the regulation of the GPX gene. Recently, Nawaz et al. [47] observed that the relative expression of GPX was higher when plants were subjected to vanadium stress. We can tentatively conclude from these studies that the expression of the GPX gene is higher when plants are treated with melatonin; however, the number of studies addressing this remains very limited.

These findings suggest that pre-treatment with melatonin can enhance the levels of functional GPX activity at the cellular level under various abiotic stresses. However, to date, only one study has reported this in the roots [31]. Furthermore, a more comprehensive analysis is required to determine the genome-wide expression of GPX genes because most plant species have more than one orthologous gene for this enzyme. Thus, further studies are needed to fully understand this influence in different parts of the plant and under high alkaline pH and heavy metal stress. 

## 6. Exogenous Melatonin and Enzymes Involved in the Ascorbate–Glutathione Cycle

**Figure 2:**
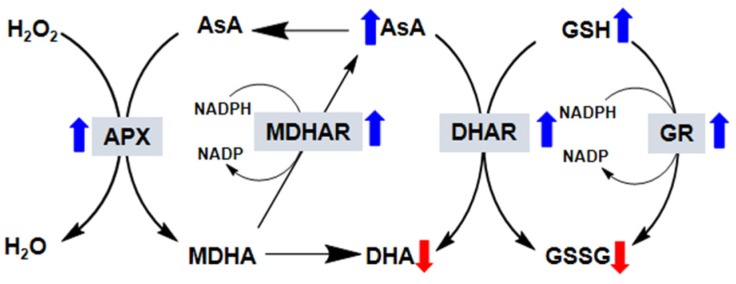
Effect of exogenous melatonin on the ascorbate–glutathione (AsA-GCH) cycle in plants. In this reaction, AsA in its reduced form is oxidized to monodehydroascorbate (MDHA). MDHA is then either reduced by monodehydroascorbate reductase (MDHAR) to AsA or, because it is very unstable, reacts to form dehydroascorbate (DHA). DHA is reduced by dehydroascorbate reductase (DHAR) to AsA. In this reaction, the reduced form of glutathione (GSH) is oxidized to glutathione disulfide (GSSG). GSSG is then reduced by glutathione reductase (GR) to GSH. The electron acceptor nicotinamide adenine dinucleotide phosphate (NADP) is regenerated during the reduction of MDHA and GSSG by the respective enzymes. AsA and GSH are also able to detoxify reactive oxygen species via direct chemical interaction. Thus, in addition to the total AsA and GSH levels, their redox state (i.e., reduced vs. oxidized), which depends on the activity of the four enzymes (gray boxes), is also very important for successful plant responses to stress. The blue arrows indicate that the exogenous application of melatonin increases the activity of these enzymes, whereas the red arrows indicate the opposite.

Changes in the AsA to dehydroascorbate (DHA) ratio and the GSH to glutathione disulfide (GSSG) ratio are used to sense oxidative stress in plant cells and generate a response. This AsA-GSH cycle is also known as the Halliwell–Asada pathway, and it plays an important role in AsA and GSH regeneration and in detoxifying the cell of H_2_O_2_. The AsA-GSH conversion cycle involves the step-wise oxidation and reduction of GSH, AsA, and nicotinamide adenine dinucleotide phosphate (NADPH), which is catalyzed by the enzymes DHAR, APX, MDHAR, and GR, and occurs in four subcellular locations: the chloroplasts, peroxisomes, cytosol, and mitochondria. The cycle has a key role in overcoming the oxidative stress induced by environmental stress [8]. The four enzymes that take part in the AsA-GSH cycle are of extreme importance to the tolerance of plants to oxidative stress by maintaining high AsA/(AsA + DHA) and GSH/(GSH + GSSG) ratios [112]. The effects of exogenous melatonin on the activities of four enzymes are discussed in this section (Figure 2).

### 6.1. Ascorbate Peroxidase (APX)

APX (EC 1.11.1.1) is the primary enzyme involved in the AsA-GSH cycle, playing a pivotal role in controlling internal ROS levels. Two molecules of APX are used to reduce H_2_O_2_ to water with the continuous generation of MDHA. APX is a member of the class I superfamily of heme peroxidases regulated by redox signals and H_2_O_2_ [66]. On the basis of its sequence of amino acids, five chemically and enzymatically distinct APX isoenzymes are present in subcellular compartments: stromal, cytosolic, thylakoidal, peroxisomal, and mitochondrial isoforms [113,114]. APX enzyme is found in organelles performing H_2_O_2_-scavenging within organelles. The cytosolic and chloroplastic APX isoforms are specific for AsA as electron donors, unlike the cytosolic versions, which are less sensitive to AsA depletion than the chloroplast enzymes such as stromal and thylakoid-bound enzymes [115]. When the plant is exposed to stressful situations such as cold, salt, heat, physical injury, pathogens, and drought, APX levels begin to increase. However, enzyme biosynthesis differs between the various subcellular locations and also depends on the plant’s developmental stage and the stress conditions. Enzyme activity is enhanced along with that of other antioxidants, which work in conjunction with APX [116,117]. 

The application of melatonin to crops under various abiotic stresses (both individual and combined) has been reported by several studies. Li et al. [14] found that APX activity increased in *Citrullus lanatus* L. under NaCl stress only when seedlings were pre-treated with a concentration of 150 μM but not 50 μM. Similarly, under salinity stress, greater APX activity has been reported by Jiang et al. [18], Wang et al. [19], Li et al. [20], Siddiqui et al. [23], and Gao et al. [24] in *Zea mays* L., *Cucumis sativus* L., *Malus hupehensis* L., *Solanum lycopersicum* L., and *Avena nuda* L., respectively. Campos et al. [25], Ye et al. [26], Liu et al. [30], Wang et al. [27], and Li et al. [29] have also reported enhanced APX activity following treatment with exogenous melatonin at concentrations ranging from 20 to 100 μM under drought stress. Similarly, higher production of APX has been reported under cold stress, high light stress, cold-induced damage, and heavy metal stress by Li et al. [33], Lee and Back [48], and Ding et al. [35], respectively. Furthermore, Martinez et al. [50] described the effects of exogenous melatonin on APX activities under combined stresses (salinity + heat, 35 °C + 75 mM NaCl) in *Solanum lycopersicon* L and found that, similar to the individual abiotic stresses, the application of melatonin (100 µM) under combined stresses also increased the production of APX. These studies suggest that melatonin positively regulates APX production under both individual and combined abiotic stresses. 

Under both normal and extreme conditions, H_2_O_2_ is produced within the cytosol and organelles. To maintain normal levels of H_2_O_2,_ the expression of the various APX isoforms occurs in the cell. Nawaz et al. [47] measured the transcript levels in *Citrullus lanatus* L. seedlings pre-treated with melatonin and exposed to vanadium stress. They found that the relative abundance of APX transcripts increased in these seedlings compared to those not treated with melatonin. Cao et al. [52] subsequently found that APX gene expression is enhanced in *Prunus persica Batsch* L. Hujing pre-treated with melatonin under oxidative stress, and Ni et al. [38] found a clear effect on the transcript levels of APX under oxidative stress. In addition, Martinez et al. [50] reported the higher expression of APX in *Solanum lycopersicon* L. under combined stresses (salinity + heat, 35 °C + 75 mM NaCl). Thus, it can be seen that pre-treatment with melatonin boosts the expression of the APX gene in different crops under various harsh conditions.

From the available literature, we can conclude that the exogenous application of melatonin to plants positively effects on APX activities under various abiotic stresses. However, limited information is available on the melatonin and APX activities under combined stresses, and the effect of melatonin on the different APX isoforms both in the cytosol and organelles needs to be explored. Thus, to further the current understanding of the effect of melatonin on APX, additional research is needed.

### 6.2. Monodehydroascorbate Reductase (MDHAR) and Dehydroascorbate Reductase (DHAR)

MDHAR (EC 1.6.5.4) is responsible for the regeneration of AsA in cells from short-lived MDHA with the use of NADPH as a reducing agent. Because this step regenerates AsA, the enzyme is co-localized with APX in the mitochondria and peroxisomes, where the enzyme scavenges peroxides, leading to AsA oxidation [66]. Many isozymes are localized in the mitochondria, chloroplasts, cytosol, peroxisomes, and glyoxysomes. DHAR reduces dehydroascorbate (DHA) into AsA, making use of reduced glutathione (GSH) as an electron donor [118,119]. This means DHAR (EC 1.8.5.1) also plays a role in the regeneration of the AsA pool. This is important in regulating the AsA pool size in symplasts and apoplasts, maintaining the redox state [119]. This enzyme is abundant in the seeds, roots, and both green and etiolated shoots. MDAR activity is upregulated under conditions of stress, such as in tomato plants cultivated in saline soil under various light intensities [120], lower temperatures [121], and ultraviolet C (UV-C) stress in *Arabidopsis* [122]. 

Many scientists have reported that the exogenous application of melatonin affects MDAR biosynthesis under many conditions. For example, under salt stress (300 mM NaCl), pre-treatment with various doses of melatonin (50, 150, and 500 μM) led to higher MDHAR and DHAR activity in plants [14]. Martinez et al. [50] also reported higher production of MDHAR and DHAR under combined salinity and heat stress. Similarly, under higher temperatures, higher MDHAR and DHAR activity has been reported in *Cucumis sativus* L. leaves. Similarly, the overexpression of the transcripts that code for MDHAR and DHAR has been observed in plants pre-treated with melatonin under various abiotic stresses [38,50,52]. A review of the currently available literature reveals that the application of exogenous melatonin positively regulates MDHAR and DHAR at both the protein and mRNA levels.

### 6.3. Glutathione Reductase (GR)

GR (EC 1.6.4.2) is another protein enzyme with a flavin group that exhibits oxidoreductase activity. This enzyme uses NADPH as a reducing agent to remove GSSG and produce GSH. Reduced GSH is employed to regenerate AA and is converted to its oxidized state GSSG. GR is an important enzyme that catalyzes disulfide formation in GSSG to maintain a beneficial cellular GSH/GSSG ratio. It is mainly present in chloroplasts, with small quantities found in the mitochondria and cytosol. The enzyme has a low molecular weight and acts as a reducing agent to prevent thiol oxidation and reacts with harmful ROS such as O_2_^•−^ and ^•^OH [51,66].

It is evident from the literature that the activity of GR is differentially modulated by the application of exogenous melatonin under various stresses. In this context, both GR and melatonin have been reported to play crucial roles in determining the stress tolerance of plants. For instance, [30] reported the higher biosynthesis of GR in melatonin-treated *S. lycopersicum* L. under drought stress. This treatment increased the NADP^+^/NADPH ratio, boosted the supply of NADP^+^, and also helped to maintain a high GSH/GSSH ratio, which is associated with photosynthetic electron transport. Recently, similar findings were reported for maize seedlings under drought stress [31]. Turk et al. [32] also reported that GR activity increased in *Tiritucum aestivum* due to cold stress, which was further increased in seedlings pre-treated with melatonin. Subsequently, higher GR activity was reported under heat stress by [37] and [22] in *Actinidia deliciosa* and *Zea mays* L., respectively. Their findings suggest that pre-treatment with melatonin increases GR activity, which contributes to the AsA-GSH cycle and consequently helps plants under stress conditions to maintain a high AsA to GSH ratio. In addition, Martinez et al. [50] studied melatonin and GR activities under combined stresses (salinity and heat), noting greater GR activity in melatonin-treated tomato seedlings. Furthermore, higher GR activity has been found at mRNA levels by Cao, et al. [52], Martinez, et al. [50] and Ni, et al. [38]. 

On the basis of the available literature, it is apparent that the exogenous application of melatonin increases the activity of GR at both the protein and mRNA levels in various crops under extreme conditions. However, further research is needed to reveal the effects of melatonin on GR activity in the different parts of a plant.

## 7. Melatonin and Non-Enzymatic Antioxidants in Plants

To counteract ROS, plants employ a non-enzymatic detoxification system consisting of phenolic compounds, flavonoids, carotenoids, GSH, AsA, alkaloids, α-tocopherol, and some amino acids as non-enzymatic defense molecules. Details of the non-enzymatic machinery and its effects by exogenous melatonin are discussed in this section. AsA, one of the most studied and most powerful antioxidant compounds [123,124], is usually synthesized in the Smirnoff–Wheeler pathway and catalyzed by L-galactano-γ-lactone dehydrogenase in the mitochondria of the plant cell [51]. However, the translocation of AsA takes place in other cell compartments. As with other scavengers such as melatonin, AsA can also directly scavenge ROS in the cell. The enzyme is oxidized in two steps, starting with the oxidization of MDHA, which is not reduced to AsA immediately but is instead disassembled into DHA and AsA. AsA reacts with hydrogen peroxide, superoxide anion, and hydroxyl ions, leading to the regeneration of α-tocopherol from tocopherol radicals, thus protecting the membrane from oxidative damage [125]. It is also linked with the de-epoxidase enzyme violaxanthin and acts as a response matrix for APX [126].

GSH, a water-soluble and a thiol tripeptide (γ-glutamyl-cysteinyl-glycine) antioxidant compound with low molecular weight, scavenges H^2^O^2^, ^1^O_2_, OH•, and O•^−2^ [101,127]. GSH defends thiol groups present in enzymes localized in chloroplast stroma and takes part in α-tocopherol and ascorbate production [124,128]. GSH also plays a vital role in regenerating AsA to yield GSSG. The conversion of GSSG to GSH occurs either enzymatically via GR or by de novo synthesis, ultimately replenishing the cellular GSH pool. Carotenoids are phenolic compounds found in a large variety of fruits and vegetables [90] that can prevent lipid peroxidation by scavenging singlet oxygen from chloroplasts [129]. These compounds are produced in plastids and contain 40-carbon isoprenoids. Carotenoids can be classified as carotenes, which include hydrogen and carbon, and xanthophylls, which are oxidized versions of carotenes [130]. The (α, β, γ, and δ) tocopherols containing a chromanal ring structure and a phytyl tail and having different number and position in the methyl group in the ring. Tocopherols compounds are lipid-soluble and have pronounced antioxidant properties. They react more rapidly than polyunsaturated fatty acids with peroxyl radicals and hence act to break the chain reaction of lipid peroxidation [131].

Due to its fluidity, α-tocopherol can move freely within the lipid membrane, thus providing membrane protection [124,132]. Furthermore, some amino acids such as proline also act as powerful non-antioxidant compounds, counteracting the damage caused by excessive ROS. For example, it has been reported that the accumulation of proline in plants is higher under harsh conditions, either due to greater synthesis or reduced degradation [51,133].

Chen et al. [22] reported that, when maize seedlings were subjected to high salinity and treated with melatonin, relatively high levels of AsA and GSH were observed. However, the levels of DHA and GSSG were quite low compared to non-treated sprouts. Likewise, Li et al. [14] found that melatonin pre-treatment led to elevated levels of GSH and AsA but lower GSSG and DHA levels under salt stress. The GSH/GSSG and AsA/DHA ratios were also greater in plants pre-treated with melatonin compared to the controls after NaCl stress. Furthermore, higher levels of AsA and GSH have also been described in other crops following treatment with melatonin under salinity stress [19,23,50]. The higher production of AsA in response to drought conditions in tomato plants was reported by Liu et al. [30], and AsA and DHA levels increased significantly in the leaves and roots in maize stressed with drought. Huang et al. [31] described that the AsA and DHA level increased in the leaves and roots when maize seedling was irrigated with melatonin. However, spraying the leaves with melatonin did not significantly change the AsA and DHA levels, except 100 μM melatonin reduced their levels in the roots. Huang et al. [31] also described that root irrigation with melatonin could improve the level of GSH and lower the GSSG content, but leaf spraying had no influence on GSH and GSSG in leaves. Overall, melatonin application raises AsA and GSH levels following drought exposure [31], and similar findings have been reported by Shi, et al. [94], Wang, et al. [27], and Ding, et al. [94]. Similarly, the higher production of GSH and AsA during the heat and cold stress was reported by Liang et al. [37] and Li et al. [33], respectively. In addition, increased production of AsA and GSH and lower GSSG and DHA levels have been reported in several studies [23,33,34,35,38,50].

These data indicate that melatonin might have an important effect on non-enzymatic antioxidants, which could subsequently alleviate the damage caused by various abiotic stresses. However, although the effect of exogenous melatonin on non-enzymatic antioxidants has been described in detail, comprehensive research is required to explore this under a combination of various abiotic stresses. In addition, further studies are needed to examine the basic mechanisms underlying exogenous melatonin and non-enzymatic antioxidants activities.

## 8. Future Prospects

It is well-known that abiotic and biotic stress increases the production of ROS, which ultimately leads to oxidative stress in various plants. Higher concentrations of ROS negatively affect cell membranes and lead to the oxidative damage of macromolecules, rendering them nonfunctional. However, plant cells and their organelles have numerous non-enzymatic or enzymatic antioxidant mechanisms that can defend against ROS-induced oxidative pressure and thus prevent or alleviate detrimental ROS effects. Melatonin is multifunctional in terms of its role in plant growth, plant development, and the stress response. It acts as a scavenger to reduce ROS, H_2_O_2_, O_2_^-^, and OH^•^ directly and also plays a role in regulating factors related to the activity of antioxidant enzymes. The studies conducted to date suggest that melatonin application increases the stress tolerance of crops by regulating the antioxidant defense system to restore plant growth by decreasing lipid peroxidation, maintaining membrane integrity, and decreasing plasma membrane permeability. Further investigation should focus on clarifying how melatonin functions as an integrated factor. In addition, though melatonin and nitric oxide are currently investigated in some aspects, the search for diverse biological functions and unexpected regulations of both plants can be of great interest to biologists and physiologists in order to investigate experimental evidence for stress tolerance. The signaling role of these two molecules’ nitric oxide (NO)-melatonin was expected to be involved in many long-distance cross-talks with other NO conjugates such as S-Nitrosylation (SNO) and S-Nitrosoglutation (GSNO). The application of nitric oxide and melatonin biosynthesis pathway can reveal the change related with the formation of NO-melatonin in plants. NO and melatonin application can change the separate use of nitric oxide donor, whereas for melatonin this remains unanswered, and thus further investigation should be made from this perspective. These NO-based studies have to be integrated with receptors’ responses. There are at least studies on the receptors of melatonin, though most have been performed in mammals; however, a sole report of phytomelatonin Candidate G-Protein Coupled Receptor 2 (CAND2/PMTR1) in *Arabidopsis* has been reported [8].

The effect of melatonin application depends on the plant species, application time, and what parts of the plant are affected. It should be noted that most of the results reported in this review come from hydroponic experiments and that field trials remain scarce. Although important advances have been made in recent years, gaps remain in the understanding the influence of exogenous melatonin and plants’ antioxidant machinery. How the tradeoff occurs during stress, endogenous melatonin synthesis, and responses of antioxidant setup have been little perceived. Advanced imaging techniques confocal microscopy and utilizing mutant model plants could usher various underlying pitfalls of melatonin signaling. Other recent approaches such as transcriptomics, proteomics, metabolomics, and genome editing technologies such as clustered regularly interspaced short palindromic repeats and CRISPR-associated protein 9 (CRISPR/Cas9) will also offer detailed insight into melatonin biosynthesis and functional role under harsh conditions and its impact on antioxidant apparatus. It is also clear that a transgenic approach that leads to antioxidant overexpression could possibly lead to higher plant tolerance to multiple stresses via activating the melatonin metabolism.

## Figures and Tables

**Table 1:** Role of melatonin in oxidative stress in various crop plants and its regulation of various antioxidants.

Crop Plant	Stress Condition	Exo-Melatonin Based Up-Regulated Antioxidants	Exo-Melatonin Based Down-Regulated Antioxidants	Exo Melatonin Based Variable Antioxidants	References
*Zea mays* L.	Salinity	POD, APX			[18]
*Cucumis sativus* L.	Salinity	SOD, POD, CAT, APX, ASA, GSH			[19]
*Malus hupehensis Rehd.*	Salinity	APX, CAT, POD			[20]
*Cucumis sativus* L.	Salinity	CAT, SOD, POD			[21]
*Citrullus lanatus* L.	Salinity	GSH, ASA, CAT, APX, DHAR, MDHAR	GSSG, DHA		[14]
*Zea mays* L.	Salinity	APX, CAT, POD GPX, SOD, GR, GSH, ASA	GSSG, DHA		[22]
*Solanum lycopersicum* L. cv	Salinity	SOD, CAT, GR, APX, GSH, ASA	GSSH, DHA		[23]
*Avena nuda* L.	Salinity	SOD, POD, CAT, APX			[24]
*Coffea arabica* L.	Drought	CAT, APX		SOD	[25]
*Zea mays* L.	Drought	SOD, CAT, APX, POD			[26]
*Malus domestica* Borkh.	Drought	SOD, POD, APX, GSH			[27]
*Festuca arundinacea Schreb.*	Drought	CAT, POD			[28]
*Brassica napus* L.	Drought	POD, CAT, APX			[29]
*Solanum lycopersicum* L.	Drought	SOD, CAT, APX, GR, ASA		POD	[30]
*Zea mays* L.	Drought	CAT, SOD, APX, GPX AsA, DHA, GSH		GR, GSSG	[31]
*Tiritucum aestivum* cv.	Cold Stress	SOD, GPX, APX, GR		CAT	[32]
*Camellia sinensis* L.	Cold stress	APX, CAT, SOD, GR, GSH	GSSG		[33]
*Oryza sativa* L.	Cold stress	SOD, CAT, POD, GSH			[14]
*Cucumis sativus* L.	Cold stress	SOD, GSSG		CAT, POX	[34]
*Solanum lycopersicum* L.	Cold stress	SOD, CAT, APX, POD, ASA, GSH			[35]
*Zea mays* L.	Heat stress	GPX, GR, CAT, ASA, GSH			[36]
*Actinidia deliciosa*	Heat stress	SOD, POD, GR, ASA, GSH		CAT	[37]
*Triticum aestivum* cv.	Heavy metals	SOD, APX, GSH	CAT, POD		[38]
*Nicotiana tabacum* L	Heavy metals	SOD, APX, CAT			[39]
*Melissa officinalis* L.	Heavy metals			SOD	[40]
*Valeriana officinalis* L.	Heavy metals			SOD	[40]
*Cynodon dactylon* L.	Heavy metals	SOD, POD, CAT, GR, APX			[41]
*Solanum lycopersicum* L. cv	Acid rain stress	SOD, POD, CAT, APX			[42]
*Cucumis sativus* L.	Water stress	SOD, POD, CAT			[43]
*Malus baccata* L.	Waterlogging	SOD. POD, CAT			[44]
*Pisum sativum* L.	Oxidative stress	SOD			[45]
*Malus hupehensis* Rehd.	Alkaline stress	SOD, POD, CAT			[46]
*Citrullus lanatus*	Vanadium stress	SOD, CAT			[47]
*Arabidopsis thaliana*	High light stress	APX			[48]
*Trigonella foenum graecum* L.	Lead and acid rain stress	SOD, CAT			[49]
*Solanum lycopersicon* cv.	Salinity and heat stress	CAT, APX, GR, GST, GPX MDHAR	SOD		[50]
*Cynodon dactylon* L.	Salinity, drought and cold stress	POD, SOD, CAT, GSH			[51]

**Table 2:** List of studies validating the effect of exogenous melatonin on different antioxidant enzymes at the mRNA level in various crops. The sign ↑ indicates the upregulation of the genes for the corresponding enzymes, whereas ↓indicates downregulation, ± indicates variable regulation, and = indicates no effect.

Plant Name	Gene Name	Stress Conditions	Expression	References
*Solanum Lycopersicon* L.	CAT, DHAR	Salinity and heat	↓	[50]
	Cu/ZnSOD, FeSOD, GR, GPX, GST, APX, MDHAR, DHAR		↑	
*Cucumis sativus* L.	Cu/ZnSOD, CAT, POD	Salinity	↑	[21]
	FeSOD		±	
*Camellia sinensis* L.	APX, CAT, SOD, GR	Cold stress	↑	[33]
*Avena nuda* L.	NAC, WRKY1, MYB, DREB1	Salt stress	↑	[24]
	DREB2		±	
*Citrullus lanatus*	SOD, APX, GPX, GST, CAT	Vanadium stress	↑	[47]
*Malus domestica*	APX	Oxidative stress	±	[38]
	GR		±	
	POD, MDHAR, DHAR, CAT		↑	
*Prunus persica*	SOD, CAT, APX, DHAR	Oxidative stress	↑	[52]
	APX		=	
	GR		±	
